# A Quantitative Profiling Method of Phytohormones and Other Metabolites Applied to Barley Roots Subjected to Salinity Stress

**DOI:** 10.3389/fpls.2016.02070

**Published:** 2017-01-10

**Authors:** Da Cao, Adrian Lutz, Camilla B. Hill, Damien L. Callahan, Ute Roessner

**Affiliations:** ^1^School of BioSciences, The University of Melbourne, ParkvilleVIC, Australia; ^2^Metabolomics Australia, School of BioSciences, The University of Melbourne, ParkvilleVIC, Australia; ^3^School of Veterinary and Life Sciences, Murdoch University, MurdochWA, Australia; ^4^Centre for Chemistry and Biotechnology, School of Life and Environmental Sciences, Deakin University, BurwoodVIC, Australia

**Keywords:** phytohormone, liquid chromatography-mass spectrometry, gas chromatography-mass spectrometry, metabolomics, Na^+^ exclusion, salinity stress, barley root, hydroponics

## Abstract

As integral parts of plant signaling networks, phytohormones are involved in the regulation of plant metabolism and growth under adverse environmental conditions, including salinity. Globally, salinity is one of the most severe abiotic stressors with an estimated 800 million hectares of arable land affected. Roots are the first plant organ to sense salinity in the soil, and are the initial site of sodium (Na^+^) exposure. However, the quantification of phytohormones in roots is challenging, as they are often present at extremely low levels compared to other plant tissues. To overcome this challenge, we developed a high-throughput LC-MS method to quantify ten endogenous phytohormones and their metabolites of diverse chemical classes in roots of barley. This method was validated in a salinity stress experiment with six barley varieties grown hydroponically with and without salinity. In addition to phytohormones, we quantified 52 polar primary metabolites, including some phytohormone precursors, using established GC-MS and LC-MS methods. Phytohormone and metabolite data were correlated with physiological measurements including biomass, plant size and chlorophyll content. Root and leaf elemental analysis was performed to determine Na^+^ exclusion and K^+^ retention ability in the studied barley varieties. We identified distinct phytohormone and metabolite signatures as a response to salinity stress in different barley varieties. Abscisic acid increased in the roots of all varieties under salinity stress, and elevated root salicylic acid levels were associated with an increase in leaf chlorophyll content. Furthermore, the landrace Sahara maintained better growth, had lower Na^+^ levels and maintained high levels of the salinity stress linked metabolite putrescine as well as the phytohormone metabolite cinnamic acid, which has been shown to increase putrescine concentrations in previous studies. This study highlights the importance of root phytohormones under salinity stress and the multi-variety analysis provides an important update to analytical methodology, and adds to the current knowledge of salinity stress responses in plants at the molecular level.

## Introduction

Barley (*Hordeum vulgare* L.) is the fourth most important cereal crop in the world after wheat, maize, and rice, and is widely used for food, livestock feed and brewing beer (Bengtsson, 1992; Forster et al., 2000; Shelden et al., 2016). Barley is an important research crop model with well-studied genetics and physiological characteristics and a sequenced genome (Forster et al., 2000; The International Barley Genome Sequencing Consortium, 2012). Among the cereal crops, barley has considerably higher salt tolerance and thus is regarded as a good research model to study salt tolerance (Ashraf and Harris, 2004; Munns and Tester, 2008). Soil salinity is a major environmental constraint to crop production that affects about 45 million hectares of irrigated land and costs global agriculture an estimated US$ 27.3 billion p.a. (Qadir et al., 2014).

Phytohormones are known to respond and regulate plant growth in response to environmental cues including salinity stress (Denancé et al., 2013). For example, salinity stress can increase the production of the plant stress-response hormone, abscisic acid (ABA) which causes stomatal closure in leaves, and thus restricts plant transpiration rate and salt uptake under salinity stress (Chaves et al., 2009; Zörb et al., 2013). ABA was further shown to up-regulate the gene expression of a vacuolar Na^+^/H^+^ antiporter under salinity stress (Shi and Zhu, 2002). Up-regulation of this antiporter can alleviate plant growth reduction under salinity stress through the compartmentalization of toxic Na^+^ ions. In another example, treatment with salicylic acid (SA) was shown to stimulate the photosynthetic rate and enhances salt tolerance of a variety of plants, including *Arabidopsis*, barley, and wheat (reviewed by Hayat et al., 2010). At the genetic level, salinity stress can induce the biosynthesis of stress specific phytohormones (e.g., ABA, SA and ethylene) (Xiong et al., 2002; Xiong and Zhu, 2003). Subsequently, these phytohormones initiate a second round of signaling to further amplify the high salinity signal. In the case of ABA, this signaling cascade appears to occur through increased expression of stress-related genes linked to ABA-sensitive transcription factors, such as the ABA-responsive element binding protein (Shinozaki and Yamaguchi-Shinozaki, 2007). These examples illustrate that phytohormones regulate plant growth under salinity stress through genetic and physiological processes (e.g., stomatal closure and tissue abscission). Thus, phytohormones present promising research candidates for studying salt tolerance mechanisms and potentially counteracting reduced plant growth and yield potential under salinity stress.

Despite their importance for plant growth and development, not all phytohormone species have been detected and accurately quantified yet (Umehara et al., 2008). Many phytohormones are difficult to quantify as they occur at very low concentrations in plants and belong to diverse chemical classes that are difficult to measure using a single analytical platform. Phytohormone concentrations can vary greatly between tissue types, e.g., roots compared to leaves (Barkawi et al., 2010). Thus, continuous improvements of analytical techniques for phytohormone detection and quantitation are required to study phytohormones in tissues which contain low concentrations of phytohormones. In recent years, high performance liquid chromatography coupled to tandem mass spectrometry (LC-MS) has become the most efficient method to measure phytohormones (Chiwocha et al., 2003; Ljung et al., 2010; Pan et al., 2010; Du et al., 2012; Trapp et al., 2014; Walton et al., 2015). However, most phytohormone quantification studies use plant leaf material which often contains higher concentrations of phytohormones than other tissues (Davies, 2010). Thus, while some methods exist to quantify phytohormones in other tissue types (Du et al., 2012), none so far attempted to quantify a wide range of phytohormones and their related metabolites in roots.

In addition to targeting phytohormones, metabolomics can provides an efficient approach to study complex metabolite responses in plants under salinity stress (Widodo et al., 2009; Hill et al., 2013; Shelden et al., 2016). Metabolite profiling has been applied to a variety of cereal crops (e.g., barley, wheat, and rice) to identify differences between salt-sensitive and salt-tolerant varieties (reviewed by Obata and Fernie, 2012). For example, Widodo et al. (2009) found that the salt-tolerant barley variety Sahara, which grew better under long-term salt stress in hydroponics, showed significantly increased tricarboxylic acid (TCA) cycle intermediates and metabolites associated with cellular protection under salinity stress compared with the variety Clipper which showed drastic growth reductions. Other studies on the same barley genotypes demonstrated variety-specific effects of salinity on whole root fatty acid and lipid profiles (Natera et al., 2016), while another study detected root-zone- and variety-specific (spatial) metabolic (Shelden et al., 2016) and transcriptomic (Hill et al., 2016) signatures along longitudinal axes of roots upon salt stress.

This study aims to investigate the effects of salinity stress on barley roots through the quantitation of phytohormones and polar (primary) metabolites and to define the relationships between growth performance, phytohormones and metabolites before and after salinity stress. We present a validated LC-MS quantification method that can rapidly and reliably quantify ten different phytohormones and their metabolites belonging to diverse chemical classes in barley roots. A salinity experiment was performed using six barley varieties, including five commercially relevant Australian cultivars (including Clipper) and one Algerian landrace (Sahara), grown in a hydroponics system. To monitor plant growth under salinity stress, physiological measurements including biomass, plant length, chlorophyll concentrations were recorded in addition to the phytormones quantified using LC-MS. Also, we quantified Na^+^ and K^+^ concentrations in roots and leaves to evaluate the Na^+^ exclusion and K^+^ retention. Furthermore, three main groups of primary metabolites (amino acids, organic acids, and sugars) were quantified to provide a broad view of metabolic activities under salinity stress in different varieties. Finally, all these results were compared and correlated to connect large-scale phenotypes with small-scale metabolic changes.

## Materials and Methods

### Chemicals and Reagents

Phytohormone standards and internal standards (ISTD) used for LC-MS method were purchased from various suppliers (Supplementary Table 1). All solvents used were LC-MS grade purchased from Merck (Australia). Other chemicals were sourced from Sigma Aldrich (Australia).

### Plant Growth and Harvest

Six varieties of barley (*Hordeum vulgare* L.) were selected based on their importance for production, commercially relevant traits and salt tolerant abilities (Tavakkoli et al., 2012; Kamboj et al., 2015). The following Australian barley varieties were chosen: the malting varieties Clipper, Flagship, and Vlamingh, the food variety Hindmarsh, the feed variety Mundah; and the landrace variety Sahara 3771 (Algeria, North Africa). All seeds were sourced from the Australian Centre for Plant Functional Genomics, The University of Adelaide.

Barley seeds (20 per variety) were surface sterilized in 70% ethanol for 1 min and rinsed five times with deionized water. Seeds were sterilized using 1% sodium hypochlorite for 10 min and then rinsed five times with deionized water. Seeds were imbibed in deionized water with aeration for 16 h and then transferred to moistened filter paper for vernalization at 4°C. After 2 days, seeds were transferred to a plant growth chamber (Fitotron, Weiss Gallenkamp, UK) for four days with the temperature set to constant 17°C. After germination, seedlings were transplanted into a hydroponic system as previously described (Shavrukov et al., 2012). Seedlings were distributed randomly to avoid systematic growth environment errors. The nutrient solution was a modified Hoagland’s solution with pH adjusted to 6.0–7.0 (Genc et al., 2007). The nutrient solution was replaced weekly to reduce microbial contamination and to avoid nutrient depletion. Salt treatment was started 17 days after germination (when the second leaves had fully developed and the third leaves had just emerged) and was performed in three 25 mM NaCl increments per day until a concentration of 150 mM NaCl was reached (Genc et al., 2007). A supplement of 3.8 mM CaCl_2_ was added to the nutrient solution to maintain free Ca^2+^ levels for salt treated plants (Tester and Davenport, 2003).

All plants were harvested in a single day after 4 weeks of salt treatment. Plants were divided into two groups: 60 plants (five replicates per variety and treatment) were harvested for physiological and elemental measurements and an additional set of 60 plants for metabolite and phytohormone measurements. Plants were separated into shoots and roots. The roots in the first group were quickly rinsed (<10 s) in distilled water to remove ions on the surface for elemental composition analysis. Then, these samples were blotted dry and stored for physiological measurements and elemental composition analysis. Barley roots for metabolite and phytohormone measurements were immediately snap frozen in liquid nitrogen. All frozen samples were stored in -80°C until required.

### Growth Measurements

Shoot and root fresh weights (RFW) were measured immediately after harvest using a digital electronic balance (BW 420H, Shimadzu Corporation, Japan). At the same time, shoot and root length (RL) were measured. Then, these tissues were dried in an oven at 70°C for 48 h for dry weight measurements. Chlorophyll content was measured weekly during the salt treatment period over 4 weeks. These measurements were conducted using a SPAD chlorophyll meter (SPAD-502, Minolta, Tokyo, Japan), taken midway on the second leaf for all plant samples including control and salt treatment groups. Chlorophyll content was expressed as relative SPAD meter values (Uddling et al., 2007).

### Elemental Analysis

#### Sample Digestion for Elemental Analysis

The sample digestion method was modified from Callahan et al. (2016). The oven dried fourth leaves, as well as the roots, were individually weighed (10 ± 0.5 mg) into Eppendorf tubes containing one 3 mm tungsten carbide bead. Samples were then homogenized using a QIAGEN tissue lyser II (Qiagen, Valencia, CA, USA) three times for 30 s at 30 Hz/s. Beads were removed, then 300 μL HNO_3_ (70%) was added to each homogenized plant sample for acid digestion. Samples were placed on a thermal shaker at 1000 rpm at 70°C for 90 min. After cooling, samples were transferred to 10 mL acid washed volumetric flasks then diluted to volume with deionized water (1 mg mL^-1^). Samples were then centrifuged at 4,000 *g* for 10 min and a final dilution was made in deionized water (0.1 mg mg L^-1^).

#### Instrument Setup

Sodium and potassium quantitation was carried out using a NexION 350X ICP-MS (PerkinElmer) equipped with a SeaSpray nebulizer, cyclonic spray chamber and auto-sampler (ESI SC2-DX). Samples were introduced to the ICP nebulizer using a peristaltic pump at 20 rpm with 0.25 mm i.d. polypropylene tubing. The torch position, nebulizer gas flow rates and MS parameters were optimized using the manufacturer’s Setup solution and daily tune function (Perkin Elmer). A second sampling probe was used to provide a constant concentration of a 250 ppb scandium as the internal standard. The solutions from the sampling probe and internal standard were mixed using a mixing block prior to nebulization. Sample uptake was carried out at 48 rpm for 55 s followed by a read delay time of 15 s then an analysis at 10 rpm followed by a 30 s wash at 48 rpm. A 50 ms dwell time was used for all elements with 20 readings and 3 replicates. Quantitation was carried out using external calibration standards of 5, 10, 50, 100, 250, 500, 1,000, 5,000, and 10,000 ppb. Standards were diluted from a 1,000 ppm certified stock standards (PerkinElmer Pure Plus) matching the HNO_3_ acid concentration of the samples. All standards and sample signals were corrected using the internal standard. Two blank samples, one containing the same concentrated HNO_3_ as the sample and the other containing deionized water, were injected to subtract background signal.

### Phytohormone Profiling

#### Sample Extraction

Five individual barley roots per variety and treatment were used for phytohormone profiling. The extraction method for barley roots was modified from Trapp et al. (2014). For each sample, 100 mg frozen roots were weighed into a 2 mL Lysing Matrix D tube (MP Biomedicals, USA). The Lysing Matrix D tube was prewashed using 70% methanol. Subsequently, 1 mL 70% methanol containing 5 μl ISTD working solution (500 ng mL^-1^ salicylic-d_6_ acid, 100 ng mL^-1^ *trans*-cinnamic-d_7_ acid, 100 ng mL^-1^ dihydrojasmonic acid, 50 ng mL^-1^ indole-3-acetic-2,2-d2 acid, 50 ng mL^-1^ d5-*trans*-zeatin, 20 ng mL^-1^ d_6_-2-*cis*-4-*trans*-ABA, 10 ng mL^-1^ d2-gibberellin A_3_, 10 ng mL^-1^ d2-gibberellin A_4_) was added to the sample. Samples were then homogenized using a Cryomill coupled to a Cryolys cooler (Bertin Technologies, France) set to -10°C (6,800 rpm, 3 × 30 s, 30 s break) followed by shaking for 30 min at 900 rpm at 4°C. Then, samples were centrifuged at 15,900 rcf at 4°C for 5 min. The supernatant was transferred to a 2 mL Eppendorf tube and dried using a rotational vacuum concentrator (Christ, Germany) under full vacuum at 30°C. After that, the dried extract was reconstituted in 50 μl of starting mobile phase [5% acetonitrile (ACN) with 10 mM ammonium acetate (NH_4_Ac)] and subsequently sonicated for 10 min until the dried extract dissolved. The extract was centrifuged at 15,900 rcf at 4°C for 15 min prior to transfer to an amber vial with glass insert. Samples were stored at -80°C until LC-MS analysis.

#### Calibration Standard Sample Preparation

Ten phytohormone and related metabolite standards and eight ISTD were used for calibration (Supplementary Table 1). Standard stock solutions were prepared at 50 μg mL^-1^ and working solutions at 1 μg mL^-1^ in methanol. All stock solutions and working solutions were stored at -80°C. Calibration ranges were chosen based on the concentration of phytohormones in barley root test samples. To prepare the calibration, phytohormone standards were mixed and then serially diluted with starting mobile phase (5% ACN with 10 mM NH_4_Ac): 0, 5, 10, 20, 50, 100, 200, 400, 800 ng mL^-1^ for 12-oxo phytodienoic acid (OPDA) and SA; 0, 0.5, 1, 2, 5, 10, 20, 40, 80 ng mL^-1^ for cinnamic acid (CA); 0, 0.05, 0.1, 0.2, 0.5, 1, 2, 4, 8 ng mL^-1^ for ABA, indole-3-acetic-2,2-d_2_ acid (IAA), indole-3-carboxylic acid (ICA), gibberellin A_4_ (GA_4_), and zeatin; 0, 0.01, 0.02, 0.04, 0.1, 0.2, 0.4, 0.8, 1.6 ng mL^-1^ for jasmonic acid (JA) and gibberellin A_3_ (GA_3_). ISTD concentration was kept at a constant 100 μl mL^-1^ working solution. The calibration samples were transferred to amber vials with glass inserts and stored at -80°C for LC-MS analysis.

#### Liquid Chromatography-Mass Spectrometry

The LC-MS system was a 1290 series high performance liquid chromatograph (HPLC) and a 6490 triple quadruple (QqQ) MS equipped with a Jet Stream electrospray ionization source (AJS ESI) and an iFunnel (Agilent Technologies, Santa Clara, CA, USA). Phytohormones were separated on a Phenomenex Kinetex C18 reversed phase column (2.1 mm × 100 mm, 1.7 μm) maintained at 45°C. The mobile phases and gradient were as follows: mobile phase A: 10 mM NH_4_Ac in deionized water; mobile phase B: 10 mM NH_4_Ac in ACN. Flow rate: 0.4 mL min^-1^. The programmed step gradient was: 5% B over 0.5 min, 5–35% B over 4 min, 35–55% B over 1 min, 55–75% B over 2 min, 75–100% B over 0.1 min, followed by a clean-up step: isocratic elution at 100% B for 2 min, 100% to 5% B over 0.1 min and column wash for 2.5 min. MS parameters (for positive and negative ionization, respectively): gas temperature: 100°C; gas flow: 11 L min^-1^; nebulizer: 40 psi; sheath gas temperature: 400°C; sheath gas flow: 12 L min^-1^; capillary: ±3500 V; nozzle voltage: ±300 V; high pressure radio frequency: +120 V, -140 V; low pressure radio frequency: +80 V, -100 V. Scan type: Dynamic multiple reaction monitoring (DMRM); Q1 resolution: unit; Q3 resolution: unit. DMRM conditions for each phytohormone are listed in **Table 1**.

**Table 1:** Dynamic multiple reaction monitoring (DMRM) parameters for phytohormone standards in ammonium acetate (NH_4_Ac) mobile phase.

PH	Q1	Q3	CE	RT	SM	ISTD	Q1	Q3	CE	RT	SM
ABA	263.1	153.0	8	3.3	–	d_6_-ABA	269.2	159.2	8	3.3	–
CA	147.0	103.1	8	2.5	–	d_7_-CA	154.1	110.0	8	2.5	–
GA_3_	345.1	143.0	32	2.8	–	d_2_-GA_3_	347.1	143.0	32	2.8	–
GA_4_	331.2	243.1	16	4.5	–	d_2_-GA_4_	333.2	245.1	20	4.5	–
IAA	176.1	129.9	12	1.7	+	d_2_-IAA	178.1	132.0	12	1.7	+
ICA	160.0	115.6	12	1.8	–	d_2_-IAA	178.1	132.0	12	1.7	+
JA	209.1	59.0	8	3.5	–	H_2_JA	211.1	59.0	12	4.1	–
OPDA	291.2	165.1	16	6.7	–	H_2_JA	211.1	59.0	12	4.1	–
SA	137.0	92.9	16	1.6	–	d_6_-SA	141.0	96.9	16	1.5	–
Zeatin	220.1	135.7	20	2.9	+	d_5_-Zeatin	225.2	137.1	20	2.9	+

*PH, phytohormone; Q1, precursor ion selected in Q1; Q3, product ion selected in Q3; CE, collision energy; RT, retention time; SM, scan mode; ISTD, internal standard; ABA, 2-*cis*-4-*trans*-abscisic acid; CA, *trans*-cinnamic acid; GA_*3*_, gibberellin A_*3*_; GA_*4*_, gibberellin A_*4*_; IAA, 3-indoleacetic acid; ICA, indole-3-carboxylic acid; JA, jasmonic acid; JA-ile, jasmonoyl-isoleucine; OPDA, 12-oxo phytodienoic acid; SA, salicylic acid. d2-GA_*3*_, 17,17-d_*2*_-gibberellic acid; d2-GA_*4*_, 17,17-d_*2*_-gibberellin A_*4*_; d_*2*_-IAA, indole-3-acetic-2,2-d_*2*_ acid; d_*5*_-zeatin, d_*5*_-*trans*-zeatin; d6-ABA, d_*6*_-2-*cis*-4-*trans*-abscisic acid; d_*6*_-SA, salicylic-d_*6*_ acid; d_*7*_-CA, *trans*-cinnamic-d_*7*_ acid; H_*2*_JA, dihydrojasmonic acid.*

### Primary Metabolite Profiling

Organic acids and sugars were quantified using the GC-MS method published in Dias et al. (2015). Amino acids and amines were quantified using LC-MS as described in Boughton et al. (2011). Primary metabolites were extracted from 30 mg frozen roots using 1 mL 50% MeOH containing 4% internal standards (D-Sorbitol-^13^C_6_ /^15^N-Valine). Five hundred microliter metabolite extract were derivatized for GC-MS analysis and 100 μl extracts for LC-MS analysis.

### Data Processing and Statistical Analysis

All raw metabolite data was analyzed using Quantitative Analysis MassHunter Workstation software for QQQ (Agilent Technologies, Santa Clara, CA, USA). Outliers were excluded if the data point was outside the 1.5 × interquartile range (Miller, 1993). Minitab (Minitab Inc., State College, PA, USA) was used for statistical analysis. Statistical significance between experimental groups (treatments and varieties) was performed using Student’s t test and one-way analysis of variance (ANOVA). False discovery rate (FDR) (Hochberg and Benjamini, 1990) was used to reduce type I errors in multiple comparisons. Bar plots and line plots were created using Graph Pad Prism 6.0 (GraphPad Software, La Jolla, CA, USA). Hierarchical cluster analysis was carried out in MetaboAnalyst 3.0^1^ (Xia et al., 2015).

## Results

### Method Validation

**FIGURE 1:**
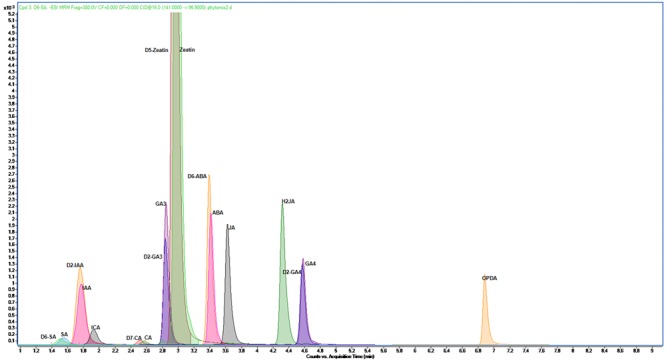
**LC-MS chromatogram showing the separation of 10 phytohormones and 8 internal standards within the standard mixture of developed method.** OPDA, 12-oxo phytodienoic acid; GA_4_, gibberellin A_4_; IAA, 3-indoleacetic acid; ICA, indole-3-carboxylic acid; ABA, abscisic acid; CA, *trans*-cinnamic acid; GA_3_, gibberellin A_3_; JA, jasmonic acid; SA, salicylic acid; Zeatin, *trans*-zeatin; d2-GA_3_, 17,17-d_2_-gibberellic acid; d2-GA_4_, 17,17-d_2_-gibberellin A_4_; d_2_-IAA, indole-3-acetic-2,2-d_2_ acid; d_5_-zeatin, d_5_-*trans*-zeatin; d6-ABA, d_6_-2-*cis*-4-*trans*-abscisic acid; d_6_-SA, salicylic-d_6_ acid; d_7_-CA, *trans*-cinnamic-d_7_ acid; H_2_JA, dihydrojasmonic acid

To validate the LC-MS methodology, the limits of detection (LOD), limits of quantification (LOQ), linearity of calibration curve, recovery and repeatability (intra-assay precision) for each phytohormone were calculated using a pooled biological quality control (PBQC) sample (**Table 2**). The PBQC was an equal mixture of extracts from six different barley varieties. LOD and LOQ were set at a signal to noise ratio (S/N) of 3 and 10, respectively. The S/N was calculated using the auto-RMS (root-mean-square) algorithm in the MassHunter Quantitative Analysis software. The LOQ for all phytohormones ranged from 0.01 to 0.9 ng g^-1^ fresh weight and LOD were in the range of 0.004–0.3 ng g^-1^ fresh weight. All calibration curves were highly linear over the calibration range with *R*^2^ ≥ 0.99. Recovery was calculated by spiking a fixed concentration of each phytohormone standard into three PBQCs. Recovery values for phytohormones ranged from 46 to 115%. The repeatability of the method was calculated as the percent relative standard deviation (%RSD) (Shabir, 2004). The % RSD values for all phytohormones were less than 0.1. A chromatogram for standards is shown in **Figure 1**.

**Table 2:** Limit of detection (LOD), limit of quantification (LOQ), linearity and repeatability for developed method.

Phytohormones	LOQ (ng g^-1^)	LOD (ng g^-1^)	Repeatability (RSD%)	*R*^2^	Recovery (%)
ABA	0.016	0.005	0.05	0.999	84.2
CA	0.465	0.140	0.04	0.999	66.6
GA_3_	0.052	0.016	0.03	0.999	90.9
GA_4_	0.102	0.031	0.09	0.998	104.3
IAA	0.882	0.265	0.06	0.999	93.1
ICA	0.866	0.260	0.02	0.999	114.6
JA	0.015	0.004	0.07	0.999	46.2
OPDA	0.043	0.013	0.04	0.999	101.2
SA	0.085	0.025	0.02	0.999	105.9
Zeatin	0.217	0.065	0.05	0.999	76.2

*RSD, relative standard deviation; ABA, 2-*cis*-4-*trans*-abscisic acid; CA, *trans*-cinnamic acid; GA_3_, gibberellin A_3_; GA_4_, gibberellin A_4_; IAA, 3-indoleacetic acid; ICA, indole-3-carboxylic acid; JA, jasmonic acid; JA-ile, jasmonoyl-isoleucine; OPDA, 12-oxo phytodienoic acid; SA, salicylic acid; *R*^2^, linear correlation coefficient.*

### Growth Performance of Hydroponically Grown Barley under Salinity Stress

#### Biomass Reduction under Salinity Stress

**FIGURE 2:**
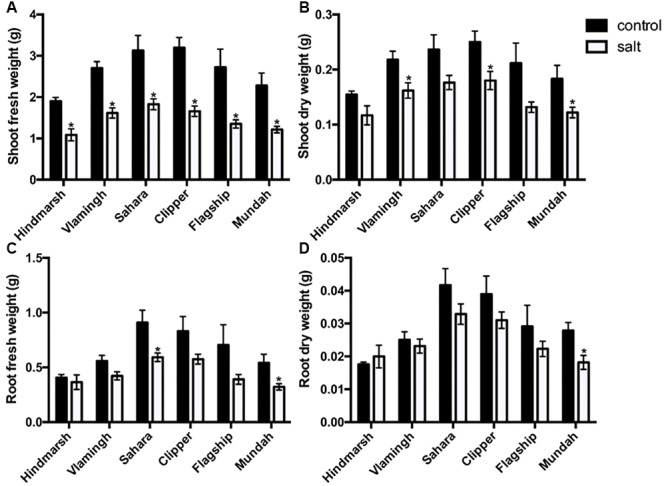
**Shoot fresh (A) and dry **(B)** weights, root fresh **(C)** and dry **(D)** weights of six barley varieties in control and salt-treated conditions (150 mM NaCl).** Data are represented as mean ± standard error, *N* = 5. Weights that are significantly (*p* < 0.05) different between control and salt treatment for each variety are indicated with asterisks.

The shoot fresh and dry weight for all varieties decreased significantly in response to 4 weeks of treatment with 150 mM NaCl (*p* < 0.05) (**Figure 2**, Supplementary Table 2). Hindmarsh, Vlamingh, and Sahara maintained the highest shoot fresh weights (SFW) (57–60%) and shoot dry weights (SDW) (74–76%). Flagship had the lowest SFW (50%) and lowest SDW (62%) compared to control conditions. However, only Vlamingh, Clipper, and Mundah showed significant decreases (*p* < 0.05) in SDW. The root fresh and dry weights also decreased under salinity stress (**Figure 2**, Supplementary Table 2). Specifically, RFW decreased between 10 and 45%, with Hindmarsh maintaining the highest RFW (90%) and Flagship and Mundah having the lowest (55–60%). However, only Sahara and Mundah showed a significant decrease in RFW under salinity stress (*p* < 0.05). Root dry weights (RDW) also decreased in some varieties after salt treatment. Hindmarsh and Vlamingh maintained the highest RDW (92–100%) and Mundah had the lowest (65%). But only Mundah showed a significant decrease in RDW (*p* < 0.05).

#### Root and Shoot Length Reduction under Salinity Stress

**FIGURE 3:**
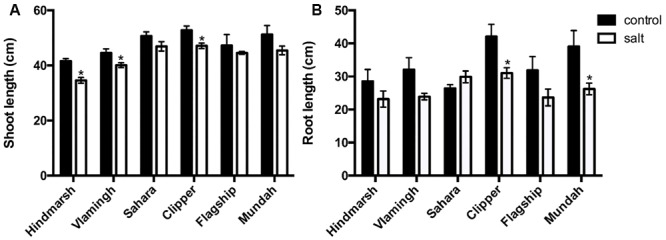
**Shoot (A) and root **(B)** lengths of barley varieties in control and salt-treated conditions (150 mM NaCl).** Data are represented as mean ± standard error, *N* = 5. Lengths that are significantly (*p* < 0.05) different between control and salt treatment for each variety are indicated with asterisks.

Root and shoot lengths (SL) were measured at the time of sample harvest. Salt treatment significantly decreased SL only in Hindmarsh, Vlamingh, and Clipper (*p* < 0.05), although a trend was observable in all varieties (**Figure 3**). Similarly, a reduction in root length (RL) was observable in all varieties except Sahara; however, this decline was only significant in Clipper and Mundah (*p* < 0.05).

#### Chlorophyll Concentrations Increase under Salinity Stress

**FIGURE 4:**
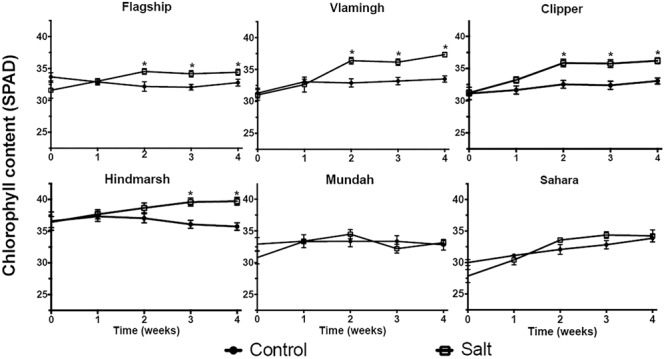
**Chlorophyll measurements of barley varieties under control and the salt-treated condition (150 mM NaCl).** Data are presented as mean ± standard error, *N* = 10. Concentrations that are significantly (*p* < 0.05) different between control and the salt treatment for each time period are indicated with asterisks.

Chlorophyll concentrations were measured weekly during the salt treatment period (**Figure 4**). Under salt treated conditions, no significant differences in chlorophyll concentrations between control and salt treated plants were detected after 1 week of exposure to 150 mM NaCl. After 2 weeks, Flagship, Vlamingh and Clipper had significantly increased chlorophyll concentrations compared to control (*p* < 0.05). After 3 weeks, chlorophyll levels had increased in all varieties and remained above controls, except for Mundah and Sahara.

### Sodium (Na^+^) and Potassium (K^+^) Levels in Leaves and Roots

Accumulation of Na^+^ in plants under salinity stress is toxic to plant growth and has an adverse effect on K^+^ accumulation which is essential for plant growth (Munns and Tester, 2008). After treatment with 150 mM NaCl, concentrations of Na^+^ increased and K^+^ decreased in barley shoots (**Table 3**). Mundah exhibited the highest Na^+^ increase (+36-fold) in shoots, whereas Sahara and Hindmarsh had the smallest increase (+22-fold and +24-fold, respectively). Shoot levels of K^+^ decreased strongly under salinity stress: Flagship had the largest K^+^ decrease (-3.4-fold), and Mundah had the smallest (-2-fold) compared to their controls. Similar to shoots, Na^+^ levels increased and K^+^ levels decreased in roots in response to salinity stress. Clipper roots maintained the smallest Na^+^ increase (+5.4-fold) and Sahara had the highest (+17-fold). K^+^ concentrations decreased significantly in the roots of all varieties (**Table 3**). Vlamingh had the highest K^+^ reduction (-3.4-fold) among all six varieties after salt treatment. In contrast, salinity stress led to the smallest K^+^ reduction (-1.7-fold) in Sahara.

**Table 3:** Elemental compositions in the fourth leaf and roots of six barley varieties in control and salt-treated conditions (150 mM NaCl).

		Hindmarsh		Vlamingh		Sahara		Clipper		Flagship		Mundah	
		Control	Salt	Control	Salt	Control	Salt	Control	Salt	Control	Salt	Control	Salt
Leaf	K^+^	60.8 ± 3.7	20.0 ± 3.3	76.7 ± 4.3	27.2 ± 5.3	64.6 ± 5.3	22.1 ± 3.0	64.1 ± 9.8	22.3 ± 2.5	80.7 ± 8.9	23.1 ± 3.6	74.8 ± 12.1	36.9 ± 3.6
	Na^+^	1.1 ± 0.2	26.0 ± 3.7	1.0 ± 0.2	28.4 ± 2.8	1.2 ± 0.2	24.2 ± 2.2	0.9 ± 0.1	27.9 ± 5.1	0.9 ± 0.1	36.8 ± 2.7	0.6 ± 0.1	21.5 ± 2.8
	K^+^/Na^+^ ratio	55.3	0.8	76.7	1.1	53.8	1.0	71.2	1.0	89.7	0.6	124.7	1.8
	Total K^+^, Na^+^	1.6 ± 0.1	1.7 ± 0.2	2.0 ± 0.1	2.0 ± 0.1	1.8 ± 0.1	1.7 ± 0.1	1.7 ± 0.3	1.9 ± 0.1	2.2 ± 0.2	2.3 ± 0.2	2.0 ± 0.3	1.9 ± 0.2
Root	K^+^	48.7 ± 3.6	25.1 ± 3.6	51.6 ± 2.2	17.3 ± 1.2	61.8 ± 2.6	41.7 ± 1.7	59.4 ± 3.5	21.1 ± 2.1	61.6 ± 2.5	28.3 ± 1.9	52.1 ± 3.0	24.8 ± 2.0
	Na^+^	3.0 ± 0.1	42.1 ± 1.2	3.2 ± 0.2	31.3 ± 2.0	1.9 ± 0.1	32.4 ± 2.6	6.6 ± 0.3	35.9 ± 1.1	3.3 ± 0.2	36.7 ± 3.0	3.5 ± 0.7	36.2 ± 1.6
	K^+^/Na^+^ ratio	16.2	0.6	16.1	0.5	32.5	1.3	9.0	0.6	18.7	0.8	14.9	0.7
	Total K^+^, Na^+^	1.4 ± 0.1	2.6 ± 0.1	1.5 ± 0.1	1.9 ± 0.1	1.8 ± 0.1	2.5 ± 0.2	1.9 ± 0.1	2.2 ± 0.1	1.9 ± 0.1	2.5 ± 0.2	1.6 ± 0.1	2.3 ± 0.1

*Data points represent as mean ± standard error, *N* = 5. Scale: K^+^ and Na^+^ in g kg^-1^, total K^+^, Na^+^ in mol kg^-1^. Values with significant differences (*p* < 0.05) compared with control are highlighted in gray.*

As a result of the general Na^+^ increase and K^+^ decrease, K^+^/Na^+^ ratios of all varieties decreased significantly in shoot and roots in response to salinity stress (**Table 3**). In shoots, Mundah maintained the highest K^+^/Na^+^ ratio (1.8) among all varieties as it had the lowest reduction in K^+^ levels after salt treatment. In contrast, Flagship had the lowest K^+^/Na^+^ ratio under salinity stress due to the highest Na^+^ accumulation among all varieties. In roots, Sahara maintained the highest K^+^/Na^+^ ratio (1.3) due to the highest K^+^ and second-lowest Na^+^ accumulation. In contrast, Vlamingh had the lowest K^+^/Na^+^ ratio (0.5) in roots. Interestingly, summing K^+^ and Na^+^ ion concentrations, barley shoots in all varieties maintained similar total molar concentrations of K^+^ and Na^+^ as the respective controls, while roots exhibited a significant increase in the sum of K^+^ and Na^+^ ions after salt treatment (**Table 3**).

### Root Phytohormone Concentrations in Response to Salinity Stress

**FIGURE 5:**
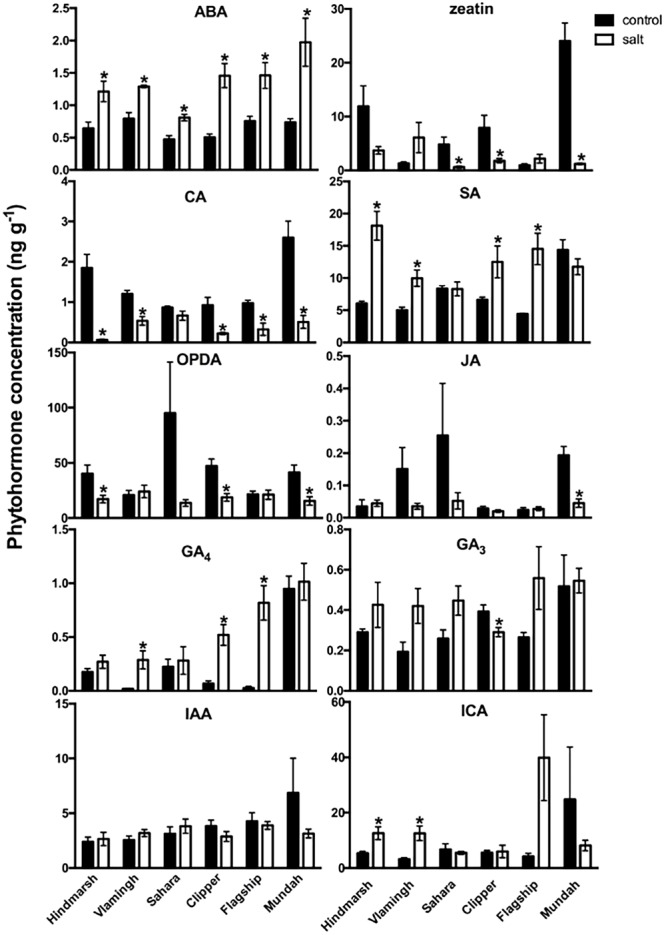
**Ten phytohormone and their metabolite concentrations (ng g^-1^ fresh weight) in barley roots under salt (150 mM) and control conditions.** Concentrations that are significantly (*p* < 0.05) different between control and salt treatment for each variety are indicated as asterisks. Data are means ± standard errors, *N* = 5. OPDA, 12-oxo phytodienoic acid; GA_4_, gibberellin A_4_; IAA, 3-indoleacetic acid; ICA, indole-3-carboxylic acid; ABA, abscisic acid; CA, *trans*-cinnamic acid; GA_3_, gibberellin A_3_; JA, jasmonic acid; SA, salicylic acid.

The LC-MS method was applied to study phytohormone levels in root tissues of six barley varieties, which differ in their responses to salinity. The concentrations of ABA, SA, CA, GA_4_, OPDA and zeatin in roots of six barley varieties changed significantly after salt treatment compared with the control condition (**Figure 5**). ABA, a well-known stress response hormone, accumulated significantly under salinity stress in all varieties (*p* < 0.05). Clipper had the strongest increase in ABA (+2.9-fold) under salinity stress compared with the other varieties (+2-fold). Changes in concentrations of SA, CA, GA_4_, OPDA and zeatin only occurred in specific varieties. Levels of SA increased significantly under salinity stress (*p* < 0.05) in four varieties including Hindmarsh (+3-fold), Vlamingh (+2-fold), Clipper (+2-fold) and Flagship (+3-fold). CA, a precursor compound for the biosynthesis of SA, decreased under salinity stress in all varieties at a range between -1.8- to -1-fold except for Sahara. GA_4_ concentrations increased strongly in the three varieties with the lowest control concentrations: Flagship (+32-fold), Clipper (+8-fold) and Vlamingh (+16-fold). Concentrations of the JA precursor OPDA (Stintzi et al., 2001) decreased significantly after salt treatment in three varieties: Mundah (-1.6-fold), Hindmarsh (-1.8-fold) and Clipper (-1.7-fold) (*p* < 0.05). JA concentrations did not change compared with control except for a decrease in Mundah (-1.2-fold). Zeatin levels significantly dropped in response to salinity stress in three varieties: Sahara (-1.1-fold), Mundah (-1-fold) and Clipper (-1.3-fold) (*p* < 0.05). Some phytohormones were mostly maintained at similar levels in control and salt treatments. The concentration of GA_3_ did not change significantly under salinity stress in any varieties except in Clipper (-3.8-fold). All six barley varieties maintained similar levels of IAA under salinity stress. Similarly, no significant concentration changes were observed for the ICA under salinity stress except for an increase in Hindmarsh (+2-fold) and Vlamingh (+4-fold).

### Metabolite Responses in Roots under Salinity Stress

**FIGURE 6:**
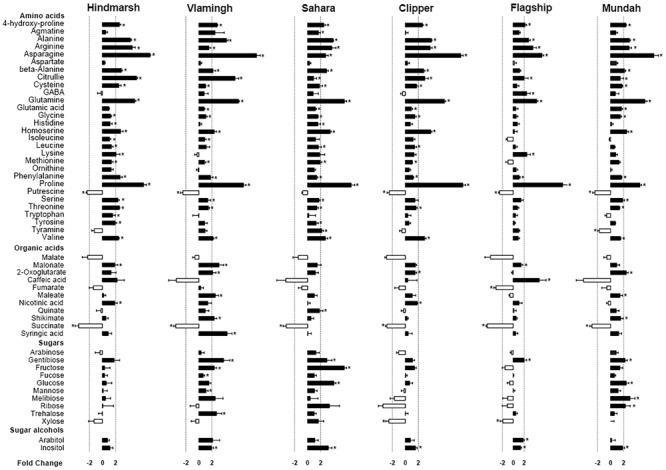
**Log_2_ fold changes of primary metabolites in roots of six barley varieties after salt treatment.** Values with significant differences compared with control (FDR adjusted *p*-value < 0.05) are indicated as asterisks. The ±2-fold change is indicated by dashed lines. Data are represented as mean ± standard error, *N* = 5.

To establish an overview of barley root metabolic activities in response to salinity stress, we measured 52 polar (mostly primary) metabolites in barley roots. Concentrations of most amino acids and amines increased significantly under salinity stress in barley roots, except putrescine levels, which decreased (-1.3- to -2.5-fold) (*p* < 0.05) (**Figure 6**, Supplementary Table 3). Specifically, eight amino acids and amines increased in all barley varieties including 4-hydroxy-proline (+2- to +3-fold), alanine (+2- to +4-fold), arginine (+2- to +5-fold), asparagine (+2- to +19- fold), citrulline (+1- to +7-fold), glutamine (+5- to +8-fold), phenylalanine (+1- to +3-fold), and proline (+9- to +27-fold). Other amino acids only showed changes in specific varieties. Hindmarsh and Sahara roots had the highest number of amino acids with changed concentrations under salinity stress with 23 and 22 changed, respectively. Vlamingh, Clipper, and Mundah had 18 compounds changed under salinity stress. Flagship showed the lowest amino acid changes with 11 compounds changing significantly (*p* < 0.05).

Only few distinct changes in organic acid concentrations after salinity stress were detected (**Figure 6**, Supplementary Table 3). Metabolites involved in the TCA cycle (succinate, 2-oxoglutarate, fumarate, maleate and malate) significantly changed in the following varieties (*p* < 0.05): Succinate significantly decreased (-1.7- to -1.3-fold) in all varieties (*p* < 0.05). 2-oxoglutarate increased significantly in Vlamingh (+2.2-fold), Clipper (+1.7-fold), and Mundah (+2.4-fold) (*p* < 0.05). Fumarate decreased in Flagship (70%). Maleate increased in Vlamingh (+2.6-fold) and Mundah (+1.7-fold). There was no significant difference in malate levels between control and salt treated barley roots. Levels of compounds involved in the shikimate pathway (shikimate, quinate) increased strongly in some varieties under salinity stress: Shikimate increased in Vlamingh (+2.3-fold) and Mundah (+1.8-fold) and quinate increased in Sahara (+2-fold). Furthermore, syringic acid and caffeic acid increased in Vlamingh (+3.6-fold) and Flagship (+6.9-fold) respectively. Nicotinic acid increased in Hindmarsh (+2-fold) and Clipper (+1.9-fold).

Changes in sugar metabolism after salinity stress were detected for several barley varieties (**Figure 6**, Supplementary Table 3). In Vlamingh, five sugars increased including fructose (+2.4-fold), fucose (+1.3 fold), gentiobiose (+4.8-fold), mannose (+1.5-fold) and trehalose (+3-fold). In Sahara, fructose (+7.3-fold), gentiobiose (+3.4-fold) and glucose (+4.1-fold) increased. In Clipper, only xylose (-1.7-fold) decreased. In Flagship, xylose (-2-fold) also decreased and gentiobiose (+2.2-fold) increased. In Mundah, gentiobiose (+2.2-fold), glucose (+2.2-fold), melibiose (+3.2-fold) and ribose (+2.5-fold) increased under salinity stress. For sugar alcohols, arabitol only increased in Flagship and inositol increased in all varieties except Hindmarsh which was the only cultivar with no significant sugar or sugar alcohol changes after salinity stress.

### Cluster and Correlation Analysis

#### Hierarchical Cluster Analysis of Metabolite Levels upon Salinity Stress

**FIGURE 7:**
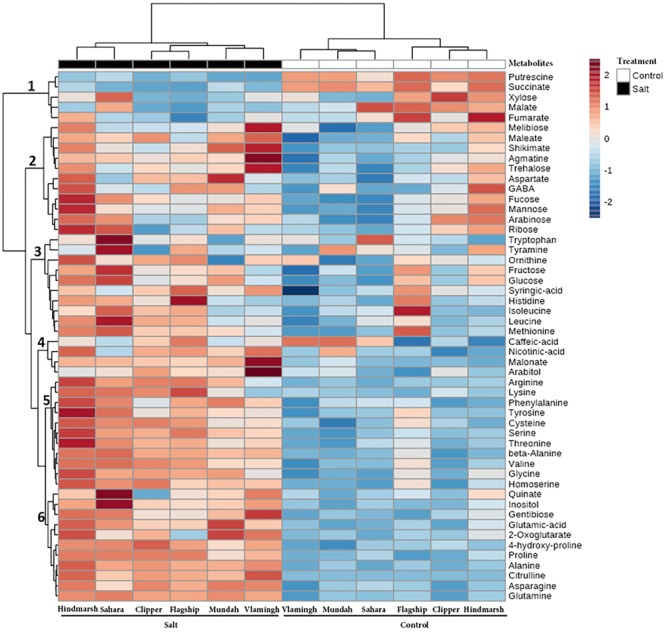
**Clustered heat map of metabolite fold changes between barley varieties under salinity stress and control conditions (Clustering Algorithm: Ward).** Clustering of the varieties and treatments is described by the dendrogram on the top. Clustering of metabolites is described by the dendrogram on the left, divided into six groups, labeled 1–6. Each colored cell represents a fold change of a metabolite average concentration (*N* = 5) before and after salt treatment.

The correlation between metabolites, varieties and treatments was examined using a clustered heat map (**Figure 7**). Overall, most metabolite concentrations increased after salt treatment compared to controls. Hindmarsh showed the highest metabolite fold changes under salinity stress among the six varieties and Vlamingh had the least changes. The hierarchical cluster analysis distinctly grouped salt treated and control plants, which exemplifies that root metabolite profiles are clearly altered by salt treatment. In the salt treated group, Hindmarsh and Sahara were grouped together and the other four varieties were clustered into a separate subgroup. However, in the control group, Hindmarsh was grouped with Clipper and Flagship, and Sahara was clustered with Mundah and Vlamingh into a separate subgroup. Different subgroups in control and salt treated groups would indicate that similarities in metabolite profiles in control conditions are not maintained after salt treatment (i.e., different varieties respond distinctly different to salt). Metabolites were grouped into six major clusters. The first cluster of metabolites primarily contains negative correlations (i.e., decrease under salinity stress) between metabolite fold changes and salinity stress, while subsequent clusters contain positive correlations (i.e., increases under salinity stress). The second cluster mostly grouped sugars together with some acids. The third and fifth clusters grouped mostly amino acids. The fourth and sixth clusters are mixed clusters with amino acids, organic acids, and sugars.

#### Correlation Analysis of Phytohormones and Primary Metabolites

Measured phytohormones were correlated with primary metabolites using Spearman rank-order correlation (**Table 4**). Only two phytohormones (CA and ABA) had strong correlations (-0.6 < ρ < 0.6) with some metabolites. CA exhibited strong positive correlations with succinate (ρ = 0.68) and putrescine (ρ = 0.68) as well as negative correlations with another 14 amino acids. ABA has strong positive correlations with citrulline (ρ = 0.69), asparagine (ρ = 0.65) and alanine (ρ = 0.62) and a negative correlation with putrescine (ρ = -0.65).

**Table 4:** Root metabolites with significant Spearman correlations to phytohormones.

Phytohormone	Metabolite	ρ	FDR adjusted *p*-value	*N*
CA	Succinate	0.68	<0.001	60
	Putrescine	0.68	<0.001	60
	Citrulline	-0.77	<0.001	60
	4-hydroxy-Proline	-0.75	<0.001	60
	Cysteine	-0.74	<0.001	60
	Threonine	-0.72	<0.001	60
	Asparagine	-0.70	<0.001	60
	Arginine	-0.70	<0.001	60
	Valine	-0.70	<0.001	60
	beta-Alanine	-0.68	<0.001	60
	Proline	-0.67	<0.001	60
	Alanine	-0.67	<0.001	60
	Serine	-0.67	<0.001	60
	Glutamine	-0.64	<0.001	60
	Phenylalanine	-0.63	<0.001	60
	Homoserine	-0.62	<0.001	60
ABA	Citrulline	0.69	<0.001	60
	Asparagine	0.65	<0.001	60
	Alanine	0.62	<0.001	60
	Putrescine	-0.65	<0.001	60

*ρ, Spearman’s rank correlation coefficient; N, number of independent samples; CA, cinnamic acid; ABA, abscisic acid; FDR, false discovery rate.*

## Discussion

### LC-MS Method for Phytohormone Quantification in Roots

The presence of many phytohormones and related metabolites is difficult to measure as they occur at very low concentrations in plants and also belong to various chemical classes (Ma et al., 2008). Quantifying phytohormones in roots is considerably more challenging than in shoots as concentrations are usually much lower (Barkawi et al., 2010). To overcome this challenge, a high-throughput LC-MS method was developed to quantify ten phytohormones and their metabolites in barley roots in 12 min. These phytohormones included JA, SA, GA, CKs, auxins, and ABA as well as some of their derivatives and precursor molecules (OPDA and CA). In this method, the sample extraction is easier without solid phase extraction and LC-MS analysis is 1.6–2 fold faster compared with previous studies (Kojima et al., 2009; Pan et al., 2010; Trapp et al., 2014). This high throughput sample extraction and analysis technique provided recovery values similar to or exceeding those previously reported (Chiwocha et al., 2003; Kojima et al., 2009; Trapp et al., 2014). The recoveries from spiked experiments were 115% (ICA), 106% (SA), 104% (GA_4_), 101% (OPDA), 93% (IAA), 90% (GA_3_), 84% (ABA), 76% (Zeatin), 66% (CA) and 46% (JA). IAA, SA, ABA, GA_3_, GA_4_, and OPDA show 1.3–5 fold higher recoveries as previous reported (Chiwocha et al., 2003; Kojima et al., 2009; Trapp et al., 2014). All % RSDs are lower than 0.1 and 100 times lower compared with RSDs reported in a previous method, which indicate the good repeatability of this quantification method (Shabir, 2004; Trapp et al., 2014). Moreover, LOQ of IAA, ABA, JA, SA, and OPDA are 2–1700 fold lower compared with Trapp’s method (Trapp et al., 2014) indicating the high sensitivity of this current method. This newly developed LC-MS method provides a fast and reliable technique to study a wide range of phytohormones in roots and could facilitate future phytohormone studies in other species under diverse environmental conditions.

### Six Barley Varieties Show Distinct Physiological Responses to Salinity Stress

#### Barley Growth Performance under Salinity Stress

Understanding plant root functions better may provide new avenues for increasing the production of important cereal and legume crops, and harnessing these root-based traits has the potential to improve crop performance under salinity stress. In this study, six barley varieties were chosen to explore the response patterns to longer-term salinity stress (150 mM NaCl for 4 weeks) in a hydroponics system, which is commonly used to assess plant salinity responses (Shavrukov et al., 2012).

Changes to agronomic and physiological characteristics such as biomass, crop yield, leaf area, plant height, chlorophyll content, and Na^+^/K^+^ ion ratio are previously reported as key indicators of salinity stress in plants (Noble and Rogers, 1992; Franco et al., 1993; Ashraf and Harris, 2004; Jamil et al., 2006; Widodo et al., 2009). In the present study, total biomass, shown to be negatively correlated with salinity stress (Moradi and Ismail, 2007), was chosen as the main growth indicator to evaluate plant performance under salinity stress in a hydroponics system. Hindmarsh was considered as the best (growth) performing variety among the six barley varieties in the hydroponics system as it maintained the largest root and shoot dry matter under salinity stress compared with its control (**Table 5**, Supplementary Table 2). Hindmarsh is a currently grown commercial Australian food variety well suited for low to medium rainfall environments with high yield potential. Our ranking results are in accordance with previous studies (**Table 5**, Supplementary Table 2) (Tavakkoli et al., 2012; Kamboj et al., 2015), with the exception of Mundah, which performed particularly poorly in our study exhibiting strong decreases in RL after salinity stress. It should be noted that Hindmarsh is a semi-dwarf barley with a shorter plant height compared to barley varieties not containing the semi-dwarfing gene (Russell et al., 2008; Jia et al., 2016). Consistent with this observation, Hindmarsh biomass (both shoot and root FW/DW) was consistently lower than other varieties in the present experiment. Previous studies determined that salt tolerant cultivars contained higher chlorophyll concentrations compared with sensitive cultivars under salinity stress (Zeng et al., 2013); however, other studies suggest that chlorophyll concentration is a poor indicator since its response is not sensitive to salinity stress (James et al., 2002). This is in accordance with results from this study, as no direct correlation between chlorophyll increase and biomass reduction could be determined. (**Figure 4**, **Table 5**).

**Table 5:** Growth performance rankings of varieties based on different growth performance indicators after salinity stress.

Variety	Ranking basis								
	Total biomass (shoot and root)	Dry weight		Na^+^ exclusion		Tissue length		Na^+^/K^+^ ratio	
		Root	Shoot	Root	Shoot	Root	Shoot	Root	Shoot
Hindmarsh	1	1	1	5	2	2	6	4	5
Vlamingh	2	2	3	3	3	3	3	6	2
Sahara	3	4	2	6	1	1	2	1	3
Clipper	4	3	4	1	4	5	4	5	4
Flagship	5	5	6	4	6	4	1	2	6
Mundah	6	6	5	2	5	6	5	3	1

#### Shoot Na^+^ Exclusion is Correlated With Plant Growth Performance under Salinity Stress

The primary sites of Na^+^ accumulation in plants are the leaves as most of Na^+^ ions are transported to the leaves from the roots with the transpiration stream of water (Munns, 2002). As a consequence, salt can accumulate to toxic levels in the leaves, reduce plant growth, and induce leaf senescence. Thus the ability of shoot Na^+^ exclusion was previously reported to be particularly crucial for crop salt tolerance (Tester and Davenport, 2003; Läuchli et al., 2008). However, Genc et al. (2015) suggested that Na^+^ exclusion is unlikely to be the main mechanism for crop salt resistance because a genetically modified wheat plant equipped with Na^+^ exclusion genes did not show higher yields under salinity. In the present study, varieties with higher biomass (Hindmarsh, Vlamingh, and Sahara) also maintained lower Na^+^ levels in shoots after salinity stress compared with varieties that were more sensitive to salinity (Clipper, Flagship, and Mundah) (**Table 3**, Supplementary Table 3). This indicates that superior shoot Na^+^ exclusion abilities are present in more tolerant varieties. No correlation between root Na^+^ levels and sensitivity toward salinity stress could be detected supporting the notion that Na^+^ accumulation and therefore toxicity may occur primarily in the leaves (Munns, 2002).

Na^+^ is known to compete for K^+^ binding sites in enzymes and also reduce K^+^ uptake and activity in plant cells (Shabala and Cuin, 2008). Therefore, the maintenance of K^+^ levels and a high K^+^/Na^+^ ratio have been suggested as potential indicators for plant salt resistance (Chen et al., 2005; Cuin et al., 2008). Other studies have tested this hypothesis but found no direct relationship between the K^+^/Na^+^ ratio and plant salt tolerance. As an example, K^+^/Na^+^ ratio was determined to be a poor predictor for the ability of barley to tolerate salinity stress (Kronzucker et al., 2006; Shelden et al., 2013). Furthermore, Genc et al. (2007) found that the K^+^/Na^+^ ratio cannot explain salt tolerance differences among 38 wheat varieties. In the present hydroponics study, there was also no direct correlation between tissue (shoot and root) K^+^/Na^+^ ratio and the biomass ranking after salinity stress (**Table 3** and **5**). The poorest performing variety Mundah even maintained the highest K**^+^** level and the highest K^+^/Na^+^ ratio in leaf. These results thus seem to confirm that the K^+^/Na^+^ ratio is not a good indicator for barley salt tolerance, at least for plants grown in a hydroponics system.

Interestingly, our study demonstrated that barley shoots in all varieties maintained similar K^+^ and Na^+^ total molar concentrations under salinity stress compared with their controls (**Table 3**). This may indicate that barley shoots accumulate predominantly inorganic ions (K**^+^**, Na**^+^**) to maintain an intracellular ionic equilibrium under salinity stress. Consequently, this ionic balance could benefit plants to cope with the osmotic stress induced by salinity stress (Munns et al., 2006). Interestingly, barley roots had increased K^+^ and Na^+^ total concentrations under salinity stress (**Table 3**). This would indicate that barley roots have different ionic balance mechanisms under salinity stress than leaves. Furthermore, this could indicate that barley roots were unable to maintain the intracellular ionic equilibrium under salinity stress and thus increased the levels of organic charged molecules (metabolites) to maintain osmotic balance (**Figure 6**).

### Phytohormone Metabolite Concentrations Differ across Six Barley Varieties in Response to Salinity Stress

The root concentrations of six phytohormone and phytohormone metabolites in six barley varieties (ABA, SA, CA, GA_4_, OPDA, and zeatin) changed significantly after salt treatment, but to different degrees. Increased levels of ABA can induce stomatal closure in leaves and reduce plant transpiration which is helpful for decreasing leaf tissue salt uptake (Zörb et al., 2013). In this study, the concentration of ABA increased significantly in roots of all barley varieties after salt treatment (**Figure 5**), irrespective of growth performance. The relationship between the increase of ABA under salinity stress and plant salt sensitivity is still not fully understood. The observed ABA accumulation may be controlled by various ABA involved activities, including ABA synthesis, catabolism, deconjugation with glucose and ABA transport between plant tissues (Verslues, 2016). Some studies suggested that plants with a lower ABA increase are more tolerant to salinity stress because high ABA accumulation is inhibitory to plant growth (Koornneef et al., 1984; Zhu, 2000). However, other studies linked increased ABA levels to lower leaf senescence rates and promotion of plant growth under salinity stress (Mäkelä et al., 2003; Munns et al., 2006). Taken together, results presented here suggest that there is no direct relationship between ABA accumulation and plant growth performance under salinity stress.

Salicylic acid is known to induce plant defense responses after pathogen infections (Malamy et al., 1990), but there is increasing evidence that SA also plays important roles to protect plants from abiotic stresses including salinity (Hayat et al., 2010). The exogenous application of SA can alleviate the negative effects of salinity stress by enhancing photosynthesis in a variety of plants including barley and wheat (Khodary, 2004; El-Tayeb, 2005; Shakirova, 2007). Specifically, SA has been linked to the synthesis of photopigments under salinity stress. For example, the exogenous application of SA increased chlorophyll concentration significantly in wheat (Hayat et al., 2005). In barley, the exogenous application of SA increased photosynthetic pigment concentrations (chlorophyll and carotenoids) and led to improved plant growth (El-Tayeb, 2005). Consistent with these findings, in the present study both SA and chlorophyll content increased concomitantly in all varieties under salinity stress (*p* < 0.05) except Sahara and Mundah (**Figure 5**) but further experimental work is required to ascertain a direct link between endogenous SA and photopigment synthesis akin to the exogenous SA application.

Although CA is one of the precursors for SA biosynthesis, no direct correlation was found for SA and CA concentration changes in the present study (**Figure 5**). A possible explanation may be that SA biosynthesis under salinity stress is mainly controlled by an alternative synthesis pathway through isochorismate. This pathway has been found to mainly regulate SA biosynthesis under UV or pathogen-stress (Wildermuth et al., 2001; Vlot et al., 2009). Furthermore, Alonso-Ramírez et al. (2009) reported that salinity stress could enhance the expression of isochorismate in *Arabidopsis* seeds. Thus, it is possible that SA is also mainly synthesized through this pathway under salinity stress in barley roots. A positive correlation was found for CA and putrescine (**Table 4**). Putrescine, which is one of the major polyamines in plants, plays important roles in plant salt resistance (Urano et al., 2004; Verma and Mishra, 2005; Gill and Tuteja, 2010). Specifically, Urano et al. (2004) reported that a salt-sensitive *Arabidopsis* mutant contained less putrescine under salinity stress and this salt sensitivity can be reversed with the addition of exogenous putrescine. The barley landrace Sahara, (**Table 5**) maintained high levels of putrescine, its precursor agmatine as well as the SA precursor CA, which has been shown to increase putrescine concentrations (Huang and Bie, 2010).

Gibberellic acids (GA) have been intensively studied and known to induce seed germination, cell elongation and cell division in plants (Schwechheimer, 2008). GA induces the degradation of DELLA protein growth repressors and thus enhances plant growth and germination, as well as flowering, and fertility (Achard et al., 2006). With respect to salinity stress, some studies suggest that GA_3_ can alleviate adverse effects by modulating SA biosynthesis (Alonso-Ramírez et al., 2009). However, in the present study, there were no significant GA_3_ changes in barley roots under salinity stress except for a decrease in Clipper (**Figure 5**). GA_4_, which is another bioactive GA (Eriksson et al., 2006), only increased in three varieties with control plants showing only very low GA_4_ concentration (**Figure 5**). However, not all of these varieties had better growth performance under salinity stress. This may indicate that different barley varieties have different GA_4_-dependent growth mechanisms under salinity stress in roots.

Jasmonic acid has mostly been studied with respect to its defense functions in plants coping with biotic stress (Anderson et al., 2004). Recent studies also found that JA is involved in aspects of plant resistance to salinity stress such as stomata closure (Walia et al., 2007; Verslues, 2016). In the present study, no significant changes to JA levels were detected except for a decrease in Mundah (**Figure 5**). Concentrations of JA measured in barley roots here were much lower (less than 0.1 ng g^-1^ FW and close to limit of quantitation; **Figure 5**) than concentrations reported for barley leaves in an earlier study (Dey et al., 2014). JA is primarily synthesized and stored in leaves, flowers and fruits (Baldwin et al., 1994; Creelman and Mullet, 1995), which would explain the large concentration differences between roots and leaves. Levels of OPDA, one of the precursors for JA biosynthesis, decreased in Hindmarsh, Clipper and Mundah after salt treatment, but did not change in the other three varieties (**Figure 5**). The concentrations of OPDA in barley roots were much higher than JA; however no direct relationship between their concentrations and salinity response could be established in this study.

Zeatin is a phytohormone known for inducing cell division. It is mainly produced in roots, then transported to shoots with the transpiration stream (Aloni et al., 2005). Yurekli et al. (2004) reported that zeatin levels decreased in a salt-sensitive bean variety but increased in a salt-tolerant bean variety under salinity stress. Fricke et al. (2006) found that zeatin decreased in the cell elongation zone in barley leaves within 20 min of salinity stress, and then increased back to the control levels. In the present study, zeatin root concentrations decreased in three barley varieties under salinity stress (Sahara, Clipper, and Mundah) while being maintained at same levels as the controls in the others (**Figure 5**). While Clipper and Mundah showed significant decreases (*p* < 0.05) in RL under salinity stress, Sahara maintained the best RL after salt treatment. This would indicate that a reduction in zeatin is not the main driver for the observed RL reduction under salinity stress.

The phytohormone IAA promotes cell division, expansion and differentiation, thus controlling root architecture, growth and development (Woodward and Bartel, 2005; Koprivova and Kopriva, 2016). It is known that salinity stress decreases IAA concentrations in leaves and leads to reduced leaf growth (Iqbal et al., 2006; Albacete et al., 2008). However, the IAA response patterns are different for roots. For example, Zörb et al. (2013) demonstrated that there is no change in IAA levels in salt resistant maize roots under salinity stress. On the other hand, Albacete et al. (2008) found that there is an increase of IAA content in tomato roots after salt treatment. In the present study, no significant IAA concentration changes were measured between control and salt treated barley roots (**Figure 5**). The maintained IAA levels in roots could result in better root growth compared with shoot growth (IAA level decreased) under salinity stress. The better growth of roots is an essential salt adaptive response in crops because less affected roots (compared with leaves) can provide sufficient water and nutrients for plant survival during salinity stress (Albacete et al., 2008; Shelden et al., 2016). ICA, which is another auxin phytohormone, plays an important role in plant pathogen defense (Gamir et al., 2012) and the ICA biosynthetic pathway was recently identified in *Arabidopsis* (Böttcher et al., 2014). In the present study, two varieties with better growth performance under salinity stress, Hindmarsh and Vlamingh, had increased ICA concentrations after salt treatment but other varieties maintained their ICA level (**Figure 5**). However, based on the limited results and the limited current knowledge, it is not possible to interpret the ICA increases in specific varieties under salinity stress.

### Metabolite Contents Differ across Six Barley Varieties in Response to Salinity Stress

Osmotic stress induced by salinity stress can lead to adverse effects on plant turgor pressure in the cell. To maintain the osmotic pressure, cells need to osmotically adjust by synthesizing compatible solutes, such as amino acids or sugars, to continue water and nutrient uptake and maintain normal growth of roots and shoots (Shelden et al., 2016). In all varieties, there were significant increases in amino acids after salt treatment (**Figure 6**). The better performing varieties Hindmarsh and Sahara, showed more increased amino acids compared with other varieties. This is likely linked to their better root growths including the higher dry matter and RL compared with other varieties (Shelden et al., 2013, 2016). Proline accumulates in several plant species under stressful environmental conditions including salt, drought, heat and cold where it mitigates the adverse effects of stress in multiple ways such as protecting cell structures, protein integrity and enhancing enzyme activities (Szabados and Savoure, 2010). In barley, proline has been demonstrated to increase in response to salinity stress in roots and shoots (Widodo et al., 2009; Shelden et al., 2016); however, often studies did not detect a relationship between proline levels and salinity tolerance (Chen et al., 2007; Shelden et al., 2016). In the present study, differences in proline accumulation are not correlated with biomass rankings (**Table 5**). This indicates that the difference of proline accumulation in roots is a poor indicator for barley salt tolerance.

After salt treatment, some organic acids decreased in barley roots. The lower concentrations of components involved in the TCA cycle under salinity stress have previously been described in other plant species, such as rice, *Arabidopsis* and grapevine (Gong et al., 2005; Cramer et al., 2007; Zuther et al., 2007). In barley leaves, Widodo et al. (2009) found that Clipper displayed a reduction of TCA intermediates with salt treatment but Sahara showed an increase. The authors suggested that lower TCA levels may be related with a reduction in metabolism in leaves, which could explain the better growth of Sahara under salinity stress compared with Clipper, when grown in hydroponics. In the present study, both Clipper and Sahara’s roots showed a similar reduction of TCA intermediates (succinate and fumarate) (**Figure 6**). This would suggest that different plant organs (shoot and root) have different metabolic activities under salinity stress and emphasizes the importance of root metabolite analyses. Furthermore, the concentration reduction of the TCA intermediates fumarate and succinate would suggest that salinity stress induces a decrease of energy generation and thus, impedes barley root growth (Zuther et al., 2007; Widodo et al., 2009).

Sugar accumulation in plants under salinity stress is known to contribute to the maintenance of osmotic pressure but is also necessary to maintain carbohydrate levels for the synthesis of cell walls (Shelden et al., 2016) and for energy consumption. Sugars also play essential roles as signaling molecules in plants after salt treatment (León and Sheen, 2003). In the present study, the change in sugar metabolism varies among different barley varieties (**Figure 6**). Fructose and glucose, which are the main monosaccharides in plants, had the highest concentration increase in Sahara roots compared with other varieties. This may indicate the different sugar metabolic adaptations with salinity stress between the landrace Sahara and cultivated barleys. Widodo et al. (2009) reported that Sahara roots exhibited a significant increase of sugars under salinity stress compared to Clipper, consistent with results presented in this study (**Figure 6**). However, compared with the other four varieties, there was no direct correlation between plant growth performance and sugar level increases highlighting that comparisons between only two varieties often allow for limited conclusions.

### Multi-Variety Experiment Provides a New Insight for Salinity Studies

In the present study, we found that six barley varieties showed distinct phytohormone and metabolite changes under salinity stress (**Figures 5** and **6**). These changes were not directly correlated with plant growth performance indicating that different barley varieties exhibited distinct phytohormone and metabolite activities under salinity stress. Thus, we conclude that the comparison between only a few varieties would not provide sufficient information for the identification of salt tolerance mechanism under salinity stress in barley.

As discussed in Section “Metabolite Contents Differ across Six Barley Varieties in Response to Salinity Stress,” our results clearly demonstrate the limitations of comparing only two genotypes which is not sufficient to draw conclusions and relationships between salt tolerance and sugar responses. Furthermore, phytohormones such as SA, CA, GA4, OPDA and zeatin exhibited different activities among barley varieties after salt treatment (**Figure 5**). For example, only Sahara maintained CA level under salinity stress. GA_4_ only increased in three varieties, with control plants showing only very low GA_4_ concentration to begin with. SA only increased in four varieties after salt treatment, which correlated with chlorophyll content changes. These variety-dependent phytohormone changes indicate that barley roots exhibited different phytohormone activities under salinity stress among different varieties from a diverse genetic background. With the contrasting change pattern of phytohormones and metabolites among barley varieties under salinity stress, it is reasonable to conclude that a multi-variety experiment comparing a range of genetic background should be conducted to provide sufficient information to identify salinity tolerant mechanisms.

## Conclusion

The data presented here adds to our current understanding of how salinity stress affects plant growth, phytohormone and plant metabolism in barley roots. To overcome the challenge of phytohormone quantification in root systems, a high-throughput LC-MS method to quantify ten phytohormones and their metabolites in barley roots was developed in the present study. This method was applied to a salinity stress experiment with six well-studied barley varieties grown hydroponically to examine phytohormone changes under salinity stress in barley roots. Firstly, shoot Na^+^ exclusion ability was correlated with plant growth performance under salinity stress. The varieties with better biomass maintenance under salinity stress also appeared to have better shoot Na^+^ exclusion ability. Secondly, distinct phytohormone and metabolite signatures due to salinity stress were identified in different barley varieties: (1) ABA, the stress response phytohormone, increased significantly in the roots of all varieties under salinity stress; (2) SA, which has known links to chlorophyll biosynthesis, increased only in varieties which exhibited an increase in chlorophyll levels under salinity stress; (3) Sahara had better biomass maintenance under salinity stress and maintained high levels of the stress-linked putrescine as well as the phytohormone CA, which has been shown to increase putrescine concentrations; (4) increased concentrations of osmoprotectants including amino acids and sugars (e.g., glucose and fructose) suggested a plant osmotic response to salt; and (5) a reduction in TCA cycle components may suggest that salinity stress decreases energy production, therefore leads to reduced plant growth. In conclusion, this study developed a high-throughput LC-MS method for phytohormone quantification in barley roots and provides new information on the abundance of ten phytohormones and their metabolites, which were correlated to primary metabolite signatures in barley roots under salinity stress. Thus, it provides important information to devise future genomics, transcriptomics and metabolomics studies on phytohormone regulation under salinity stress.

## Author Contributions

Conceived and designed the experiments: DC, AL, CH, and UR. Developed the phytohormone method: DC and AL. Performed the experiments: DC. Phytohormone and metabolite analyses: DC, AL, CH, and UR. Elemental analysis: DC and DLC. Contributed reagents/materials/analysis tools: UR and DLC. Wrote the manuscript: DC, AL, and CH. All authors revised, edited and approved the manuscript.

## Conflict of Interest Statement

The authors declare that the research was conducted in the absence of any commercial or financial relationships that could be construed as a potential conflict of interest.

## Footnotes

1 http://www.metaboanalyst.ca/
